# Low Antioxidant Status of Serum Uric Acid, Bilirubin, Albumin, and Creatinine in Patients With Benign Paroxysmal Positional Vertigo

**DOI:** 10.3389/fneur.2020.601695

**Published:** 2020-11-20

**Authors:** Ke-Hang Xie, Ling-Ling Liu, Chu-Yin Su, Xiao-Feng Huang, Bao-Xing Wu, Run-Ni Liu, Hua Li, Qing-Qing Chen, Jia-Sheng He, Yong-Kun Ruan

**Affiliations:** ^1^Department of Neurology, Zhuhai City Hospital of Integrated Traditional Chinese and Western Medicine, Zhuhai, China; ^2^Department of Nephrology, Fifth Affiliated Hospital of Sun Yat-sen University, Zhuhai, China

**Keywords:** benign paroxysmal positional vertigo, antioxidant status, uric acid, bilirubin, albumin, creatinine

## Abstract

**Objective:** To investigate the roles of serum uric acid (UA), bilirubin (BIL), albumin (ALB), and creatinine (CRE) as major intravascular antioxidants, in benign paroxysmal positional vertigo (BPPV).

**Methods:** The serum levels of UA, BIL, ALB, and CRE were retrospectively analyzed in 70 patients with new-onset idiopathic BPPV and 140 age- and sex-matched healthy controls (HCs).

**Results:** Serum UA, BIL, ALB, and CRE levels were significantly lower in the BPPV group than the HC group. Furthermore, serum levels of BIL and ALB were significantly lower in the BPPV group when compared by sex. Multiple stepwise logistic regression revealed that a reduction in serum ALB was independently related to BPPV (odds ratio = 0.688; 95% confidence interval = 0.607– 0.780). Receiver operating characteristic analyses revealed a cut-off value of 45.15 g/L for ALB with a sensitivity of 74.29% (62.97– 83.07%) and specificity of 73.57% (65.71– 80.18%).

**Conclusions:** Serum levels of UA, BIL, ALB, and CRE were lower in BPPV patients, indicating a lower antioxidant status. Furthermore, a reduction in serum ALB was independently associated with BPPV. These results provide insights into the possible roles of oxidative stress in the pathogenesis of BPPV.

## Introduction

Benign paroxysmal positional vertigo (BPPV), characterized by dizziness and vertigo, has a lifetime prevalence of more than 2.4% (1). The cause of BPPV is the detachment of otoconia (calcium carbonate crystals) that either float in the semicircular canal or attach to the cupule (2). However, at present, there are limited methods and techniques available to evaluate the condition of semicircular canals and vestibules, and the physiopathological explanations of BPPV are mainly speculative. Although most recover after positional maneuvers, up to 2/3 of patients may experience chronic, obstructive instability, dizziness, and discomfort, also known as residual dizziness (3). Meanwhile, the relapse rate of BPPV in the elderly is reportedly 23.5–50% (4). Hence, further studies of the mechanism underlying the onset of BPPV could provide new and efficient treatment regimens for residual dizziness and to decrease the recurrence rate.

It has been reported that Meniere's disease (MD) may be a systemic oxidative disorder, in which excessive free radicals and oxidative stress promote microvascular damage and participate in the development of endolymphedema (5). Recent studies have shown that some of the symptoms of BPPV are similar to those of MD and can occur at any stage, including the age of onset (6). Although it is attractive to speculate that oxidative stress plays a major role in pathogenesis of BPPV, there are few validated blood markers of the antioxidant status of BPPV.

Current evidence suggests that serum uric acid (UA), bilirubin (BIL), albumin (ALB), and creatinine (CRE) are the main non-enzymatic antioxidants in human plasma, accounting for 85% of the total antioxidant capacity (7). UA is a naturally occurring product of purine metabolism and is known as a strong scavenger of peroxynitrite. UA can exert protective antioxidant effects, inhibit inflammatory cascades, reduce blood–brain barrier permeability, and protect central nervous tissue (8). BIL was previously considered as a fat-soluble metabolite with only slight cytotoxicity, but recent studies have found that BIL is a natural antioxidant with strong antioxidant activity and an endogenous scavenger of reactive oxygen species (ROS) (8). CRE is a metabolite of creatine phosphate, which exists in skeletal muscle and is one of the components of total antioxidant determination (9). ALB is the main antioxidant molecule in extracellular fluid and can remove ROS and reactive nitrogen species (RNS) produced by various reactions (10). The serum levels of these markers can be used to predict whether oxidative stress is involved in the pathology of BPPV.

ALB and CRE levels are lower in patients with BPPV as compared to healthy controls (HCs) (11, 12). However, in BPPV, the exact relationship between UA and BPPV remains controversial (13), while that between BIL and BPPV remains unknown. Overall, previous studies have failed to comprehensively investigate the significance of these four markers (UA, BIL, ALB, and CRE) of antioxidant status in BPPV.

Therefore, the aims of the present study were to (1) test the hypothesis that serum levels of UA, BIL, ALB, and CRE are low in patients with BPPV, and (2) determine if any of these markers are protective factors in BPPV.

## Materials and Methods

### Participants

The study cohort consisted of 210 people, including 70 patients with idiopathic BPPV recruited from the Neurology Department and 140 sex- and age-matched HCs recruited from the Physical Examination Center at our hospital from January 1, 2015 to December 31, 2018.

### Inclusion and Exclusion Criteria

The diagnosis of BPPV was based on a typical history of recurrent, brief attacks of positional vertigo and positioning tests, such as the Dix-Hallpike test or roll test (2). Only patients with new-onset idiopathic BPPV were include in this study. All patients with BPPV underwent canalith repositioning maneuvers of the affected semicircular canal. Moreover, for all BPPV patients, a thorough medical history was obtained and neurological testing was performed.

The exclusion criteria were as follows: (1) hospitalization for more than 48 h after vertigo; (2) secondary factors, such as a history of head trauma and vestibular neuritis; (3) pre-existing chronic instability at the onset of BPPV; (4) severe cardiovascular disease, central nervous system disease, parathyroid dysfunction, or thyroid disease; (5) severe renal and/or hepatic impairment; (6) hypertension and/or diabetes; and (7) current infection.

### Measures

Serum levels of UA, ALB, CRE, and total BIL (TBIL) were measured using a Hitachi LST008 analyzer (Hitachi High-Technologies Corporation, Tokyo, Japan) after 8–10 h of fasting during the first 48 h after the onset of BPPV. The concentrations of alanine transaminase (ALT), aspartate transaminase (AST), fasting blood-glucose (FBG), total cholesterol (TC), triglycerides (TG), high density lipoprotein cholesterol (HDL), low density lipoprotein cholesterol (LDL), and thyroid stimulating hormone (TSH) were measured by the same method.

### Ethical Approval

The study protocol was approved by the local Ethics Review Board and conducted in accordance with the ethical standards of the Declaration of Helsinki.

### Statistical Analysis

All statistical analyses were conducted using IBM SPSS Statistics for Windows, version 24.0. (IBM Corporation, Armonk, NY, USA). All data are presented as the mean ± standard deviation (SD). To evaluate the significance of the difference between the groups, the independent-samples *t*-test was used for normally distributed data, while the Mann–Whitney *U* test was used for non-normally distributed data. Analysis of covariance was used to compare serum levels of UA, TBIL, ALB, and CRE between the BPPV and HC groups. Multiple stepwise logistic regression analysis was performed to identify predictive indicators of BPPV. The odds ratio (OR) and corresponding 95% confidence interval (CI) were calculated. On-parameter receiver operating characteristic (ROC) analysis was conducted and the area under curve (AUC) was calculated using 95% CIs. Cut-off values were calculated for each factor. A probability (*p*) value of < 0.05 was considered statistically significant.

## Results

### General Statistics

The clinical characteristics of the study participants are summarized in Table 1. As compared with the HC group, serum levels of UA (*p* = 0.021) and CRE (*p* = 0.005) were significant lower, while ALB and TBIL levels were very significantly lower (*p* < 0.001) in the BPPV group (Table 1).

**Table 1:** The clinical characteristics of the study samples.

**Variables**	**BPPV (Mean ± SD)**	**Control (Mean ± SD)**	***p-*Value**
Sex (F/M)	70 (48/22)	140 (96/44)	– – – – – –
BMI (kg/m^2^)	23.64 ± 0.42	23.42 ± 0.28	0.826
Alcohol (F/M)	16 (2/14)	24 (1/23)	0.218
Smoking (F/M)	21 (1/20)	18 (3/15)	0.314
SBP (mmHg)	123.76 ± 1.50	125.54 ± 1.03	0.958
DBP (mmHg)	80.83 ± 1.05	80.39 ± 0.70	0.619
WBC (^*^10^9^/L)	6.79 ± 0.20	6.76 ± 0.13	0.683
HGB (g/L)	133.79 ± 1.4	136.39 ± 1.13	0.204
PLT (^*^10^9^/L)	231.43 ± 6.56	225.59 ± 4.15	0.313
CRE (μmol/L)	61.19 ± 1.81	67.01 ± 1.13	0.005^**^
ALT (U/L)	18.69 ± 1.09	19.26 ± 0.74	0.341
AST (U/L)	20.51 ± 0.61	21.14 ± 0.50	0.504
ALB (g/L)	43.07 ± 0.42	46.72 ± 0.22	0.000^***^
TSH (mIU/L)	1.84 ± 0.11	1.81 ± 0.07	0.934
TC (mmol/L)	5.05 ± 0.11	4.90 ± 0.06	0.592
TG (mmol/L)	1.45 ± 0.23	1.15 ± 0.05	0.577
HDL (mmol/L)	1.42 ± 0.03	1.46 ± 0.03	0.451
LDL (mmol/L)	3.02 ± 0.09	2.83 ± 0.06	0.134
UA (μmol/L)	303.00 ± 10.12	331.86 ± 7.73	0.021^*^
TBIL (μmol/L)	9.52 ± 0.47	11.90 ± 0.35	0.000^***^
FBG (mmol/L)	5.14 ± 0.05	5.10 ± 0.04	0.584

*BMI, body mass index, defined as weight in kilograms divided by the square of height in meters; SBP, systolic blood pressure; DBP, diastolic blood pressure; WBC, white blood cells; HGB, hemoglobin; PLT, blood platelet; CRE, creatinine; ALT, alanine transaminase; AST, aspartate transaminase; ALB, albumin; TSH, thyroid stimulating hormone; TC, total cholesterol; TG, triglycerid; HDL, high-density lipoprotein cholesterol; LDL, low-density lipoprotein cholesterol; UA, uric acid; TBIL, total bilirubin; FBG, fasting blood-glucose. Values are expressed as Mean ± SD. The differences were considered significant if p-value < 0.05*.

*** *p-value < 0.001*,

** *p-value < 0.01*,

* *p-value < 0.05*.

### Subgroup Analysis by Sex

**Figure 1:**
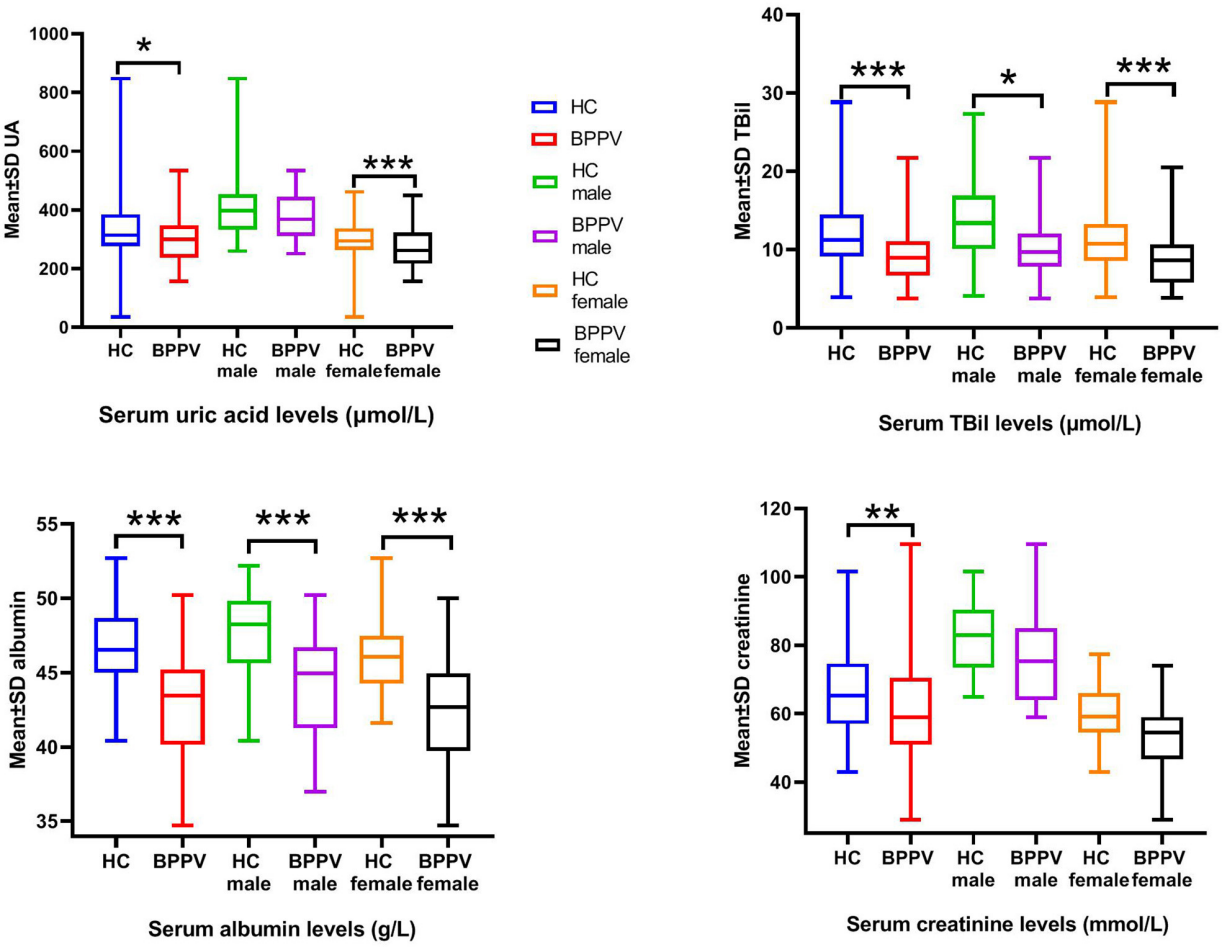
Serum levels of UA, BIL, ALB, and CRE between males and females in the BPPV and HC groups. ***p-value < 0.001, ** *p*-value < 0.01, *p–value < 0.05.

The effects of sex on serum levels of UA, TBIL, ALB, and CRE between the BPPV and HC groups were determined (Table 2, Figure 1). As expected, the results showed highly significant differences in serum levels of UA, TBIL, and CRE between males and females in the HC group, but not ALB (*p* = 0.819). In contrast, there were no significant differences in the serum levels of UA, TBIL, ALB, and CRE between males and females in the BPPV group (*p* = 0.276, 0.078, 0.826, and 0.290, respectively). Thus, there was no sex-specific differences in the BPPV group, as in the HC group. Notably, there was no sex-specific difference in serum ALB between males and females in the BPPV and HC groups.

**Table 2:** Serum levels of UA,TBIL, ALB, and CRE in the BPPV and HC groups (mean ± SD).

**Patients**	**Male**	**Female**	***P*^**1**^**	***P*^**2**^**	***P*^**3**^**
**UA**					
BPPV	371.82 ± 16.52	271.46 ± 9.80			0.276
HC	403.57 ± 15.33	299.00 ± 6.52	0.239	0.009^**^	0.000^***^
**TBIL**					
BPPV	10.73 ± 0.94	8.96 ± 0.53			0.078
HC	13.45 ± 0.71	11.19 ± 0.38	0.018^*^	0.000^***^	0.005^**^
**ALB**					
BPPV	44.07 ± 0.72	42.62 ± 0.51			0.826
HC	48.20 ± 0.37	46.04 ± 0.24	0.000^***^	0.000^***^	0.819
**CRE**					
BPPV	76.98 ± 2.70	53.95 ± 1.41			0.290
HC	82.29 ± 1.48	60.01 ± 0.79	0.392	0.096	0.000^***^

*P^1^, male patients with BPPV vs. male HC; P^2^, female patients with BPPV vs. female HC; P^3^, male vs. female in each group. The differences were considered significant if p-value < 0.05*.

*** *p-value < 0.001*,

** *p-value < 0.01*,

* *p-value < 0.05*.

Sex-specific comparisons showed that there were no significant differences in serum levels of UA and CRE among males between the BPPV and HC groups (*p* = 0.239 and 0.392, respectively). On the other hand, with the exception of CRE (*p* = 0.096), there were highly significant differences in serum levels of UA, TBIL, and ALB among females between the BPPV and HC groups. In summary, there were no significant differences in serum CRE levels between males and females in the BPPV and HC groups, but highly significant differences in serum levels of TBIL (*p* = 0.018) and ALB (*p* = 0.000) among females between the BPPV and HC groups. The high significance between males and females in the BPPV group as compared to the HC group also explains why there was no significant difference between males and females in the BPPV group.

### Multiple Stepwise Logistic Regression Analysis

Multiple stepwise logistic regression analysis unexpectedly revealed that a reduction in serum ALB, but not UA, TBIL, or CRE, was associated with BPPV (*p* < 0.05; OR = 0.688; 95% CI = 0.607–0.780) (Table 3).

**Table 3:** A multiple stepwise logistic regression to identify independent factors of BPPV.

**Variables**	**OR (95% CI)**	***p-*Value**
UA	1.000 (0.966–1.004)	0.970
TBIL	0.900 (0.818–0.991)	0.032 ^*^
ALB	0.688 (0.607–0.780)	0.000 ^***^
CRE	1.000 (0.970–1.032)	0.978

*The differences were considered significant if p-value < 0.05*.

*** *p-value < 0.001*,

*p-value < 0.01*,

* *p-value < 0.05*.

### ROC Analyses

**Figure 2:**
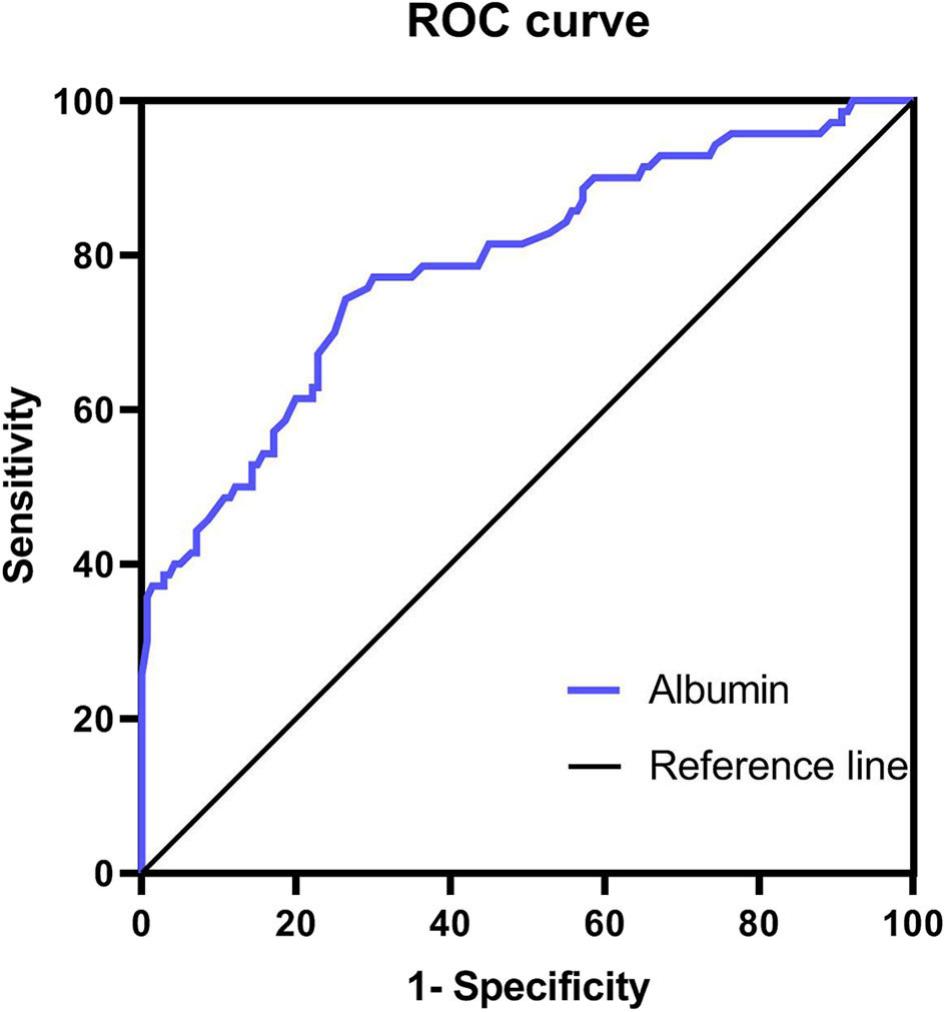
ROC curve of albumin to identify BPPV.

ROC analyses were performed to assess the levels of ALB. The AUC for ALB was 0.787 (0.720 – 0.855). The cut-off value of ALB was 45.15 g/L with a sensitivity of 74.29% (62.97– 83.07%) and specificity of 73.57% (65.71– 80.18%) (Figure 2).

## Discussion

The results of the present study showed that serum levels of UA, BIL, ALB, and CRE were significantly lower in the BPPV group than the HC group. In addition, subgroup analysis based on sex confirmed these results for BIL and ALB. Moreover, ALB was associated with BPPV. To the best of our knowledge, this is the first report of the relationship between BIL and BPPV, and the first report of UA, BIL, ALB, and CRE as indicators of oxidative stress to evaluate the antioxidant status of BPPV.

BPPV is induced by the detachment of otoconia, which are composed of inorganic calcium carbonate crystals and proteins (14). Many studies have shown that otolith shedding is associated with calcium homeostasis in the inner ear. Evidence supports a role of oxidative stress in calcium homeostasis (15). Calcium metabolism is closely related to oxidative stress (16). The endoplasmic reticulum is the main organelle for calcium storage. Under oxidative stress conditions, the endoplasmic reticulum can increase the inflow of calcium, thus, triggering ROS production and accumulation in the mitochondria (17). Subsequently, in response to reperfusion injury, calcium ions flowing into the mitochondria causes rupture of the mitochondrial membrane and subsequent apoptosis (18). Tsai et al. found that malondialdehyde levels were higher in the BPPV group before the relocation of calcium. The higher level of antioxidant superoxide dismutase in the post-treatment group suggests that oxidative stress might play a role in the pathology of BPPV (15).

In this study, serum levels of UA, BIL, ALB, and CRE were lower in the BPPV group as compared to the HC group, suggesting an association between oxidative stress and BPPV. Epidemiological studies have shown that the prevalence of BPPV is relatively high in those aged 50–60 years and the ratio of female to male is 2–3:1 (14). As shown in Figure 1, all blood markers were lower in women than men by both inter-group and intra-group comparisons (although not all findings were statistically significant), indicating weaker antioxidant capacity in women, which could explain the higher incidence of BPPV in women. Therefore, further studies are warranted to elucidate the mechanism underlying changes in serum levels of UA, BIL, ALB, and CRE associated with BPPV.

### UA and BPPV

UA is a naturally occurring product of purine metabolism and is known as a strong scavenger of peroxynitrite. In addition, UA acts as a protective antioxidant in neurodegenerative diseases, such as Parkinson's disease (19), Guillain–Barre syndrome (20), multiple sclerosis (21), and amyotrophic lateral sclerosis (22). However, there is no consensus whether UA is a protective or risk factor for BPPV. Some studies have reported that UA levels are reduced in patients with BPPV (12), while other have not replicated these findings (11). A meta-analysis reported that the relationship between UA and BPPV is complex, but may not be an independent risk factor for BPPV (13). The results of the present study indicated that UA levels were reduced in BPPV. However, there were significant differences in UA levels among females, but not males, between the BPPV and HC groups. The differences between the sexes may be caused by differences in the levels of protective estrogen in females as well as differences in dietary and lifestyle habits (23). Moreover, these differences may have something to do with the duality of UA, which is considered a natural antioxidant, but also has pro-oxidation properties, leading to increased expression of ROS, lipid peroxidation, DNA damage, and the production of inflammatory cytokines (24).

### Creatine and BPPV

CRE is a waste product produced by muscles from the breakdown of creatine and is known as an effective scavenger of free radicals (9). In this study, CRE levels were lower in the BPPV group that the HC group, which may be due to the depletion of free radicals. However, there were no significant difference in CRE levels among males and females between the BPPV groups, but not in HC groups. Actual CRE levels are easily affected by blood volume and physical condition (25). However, the lower CRE levels in the healthy females reflected lower antioxidant levels and a higher risk for BPPV.

### BIL and BPPV

Some studies have shown that BIL is an effective antioxidant even at physiological concentrations by increasing antioxidant enzyme activities and decreasing ROS levels (26). Many studies have reported a negative association between BIL levels and a range of diseases associated with oxidative stress, such as diabetes (27), asthma (28), and inflammatory bowel disease (29). Yao et al. found that short-term preservation of BIL could prevent cell damage and maintain the viability and function of transplanted islets (30). The results of the present study also revealed lower BIL levels in the BPPV group with statistical differences between males and females.

### ALB and BPPV

ALB is the major antioxidant molecule in extracellular fluid and can remove ROS and RNS. The antioxidant capacity of ALB is stronger than that of UA, BIL, ALB, and CRE (31). Redox modification changes the physiological properties of serum ALB, which can serve as a biomarker of oxidative stress (32). Actually, the oxidative state of ALB is reportedly modulated in metabolic syndrome (33), inflammation (34), and immunoglobulin A nephropathy (35). The results of the present study also revealed lower ALB levels in the BPPV group with statistical differences between males and females. Yuan et al. speculated that repeating dizziness and vomiting could lead to malnutrition and hypoproteinemia in patients with BPPV (12). We disagree with this view because relatively few patients with BPPV reported vomiting. Moreover, short-term vomiting or anorexia did not lead to a decrease in serum ALB. We believe that a low concentration of ALB represents a strong state of oxidative stress in BPPV. Furthermore, the serum ALB redox status in patients with vestibular neuritis was significantly lower than in HCs (36), as was the incidence of MD (37). Kim et al. studied the differences of protein profiles between patients with recent hearing loss and a HC group, and found that the concentrations of ALB-like proteins in the plasma and inner ear were higher in the HC group (38). In summary, ALB may be a protective factor for BPPV, although further studies are needed to clarify the underlying mechanism.

### Is BPPV an Autoimmune Disease?

Here, serum levels of BIL and ALB were decreased in the BPPV group. Multiple stepwise logistic regression analysis revealed that serum concentrations of BIL (although not statistically significant) and ALB were related to BPPV. Of note, previous studies have shown that low serum BIL and ALB levels are associated with various autoimmune diseases, such as neuromyelitis optica (39), multiple sclerosis (40), myasthenia gravis (41), and anti-N-methyl-d-aspartate receptor encephalitis (42). Hence, to determine whether BPPV is an autoimmune disease, further studies are needed to reveal the association between serum levels of BIL and ALB, and the pathogenesis of BPPV.

As discussed in previous sections, the results of the present study revealed lower serum levels of UA, BIL, ALB, and CRE in the BPPV group, suggesting higher levels of oxidative stress in BPPV patients, although it is uncertain whether low antioxidant status led to or was the result of disease. Nonetheless, the low antioxidant status in BPPV patients could not reverse damage to the vestibular system due to free radical toxicity. As another possibility, serum levels of UA, BIL, ALB, and CRE were reduced in the BPPV group because of the removal of excessive free radicals. However, future studies are needed to elucidate the pathological mechanisms underlying these associations in the inner ear.

Three were three major limitations to this study. First, this was a preliminary descriptive study, which lacked evidence of biological and pathological mechanisms. Second, only patients hospitalized for idiopathic BPPV were included in this study. Third, serum levels of UA, BIL, ALB, and CRE were not followed-up in acute and remission patients. Since data are scarce, the evidence to support the conclusion remains weak, thus further studies with larger populations are needed to define these relationships.

## Conclusions

Serum levels of UA, BIL, ALB, and CRE were reduced in patients with BPPV. In addition, reduced ALB was independently associated with BPPV, although further studies are required to clarify the underlying mechanism. This finding may be attributed to an active oxidative process in BPPV patients with low antioxidant status.

## Data Availability Statement

The data that support the findings of this study are available on request from the corresponding author. The data are not publicly available due to privacy or ethical restrictions.

## Ethics Statement

The study protocol was approved by the Ethics Committee of Zhuhai Hospital of Integrated Traditional Chinese and Western Medicine and conducted in accordance with the ethical standards of the Declaration of Helsinki.

## Author Contributions

K-HX and L-LL: conceptualization. Q-QC and HL: data curation. C-YS, X-FH, and J-SH: software. B-XW, R-NL, and Y-KR: validation. K-HX and L-LL: writing original draft and writing review and editing. All authors contributed to the article and approved the submitted version.

## Conflict of Interest

The authors declare that the research was conducted in the absence of any commercial or financial relationships that could be construed as a potential conflict of interest.
